# Classification of patient-safety incidents in primary care

**DOI:** 10.2471/BLT.17.199802

**Published:** 2018-04-23

**Authors:** Jennifer Cooper, Huw Williams, Peter Hibbert, Adrian Edwards, Asim Butt, Fiona Wood, Gareth Parry, Pam Smith, Aziz Sheikh, Liam Donaldson, Andrew Carson-Stevens

**Affiliations:** a 5th Floor Neuadd Meirionnydd, Division of Population Medicine, School of Medicine, Cardiff University, Cardiff, CF14 4YS, Wales.; b Australian Institute of Health Innovation, Macquarie University, Sydney, Australia.; c Office for National Statistics, Newport, Wales.; d Institute for Healthcare Improvement, Boston, United States of America.; e School of Health in Social Science, University of Edinburgh, Edinburgh, Scotland.; f Usher Institute of Population Health Sciences and Informatics, University of Edinburgh, Edinburgh, Scotland.; g Faculty of Epidemiology and Public Health, London School of Hygiene and Tropical Medicine, London, England.

## Abstract

Primary care lags behind secondary care in the reporting of, and learning from, incidents that put patient safety at risk. In primary care, there is no universally agreed approach to classifying the severity of harm arising from such patient-safety incidents. This lack of an agreed approach limits learning that could lead to the prevention of injury to patients. In a review of research on patient safety in primary care, we identified 21 existing approaches to the classification of harm severity. Using the World Health Organization’s (WHO’s) International Classification for Patient Safety as a reference, we undertook a framework analysis of these approaches. We then developed a new system for the classification of harm severity. To assess and classify harm, most existing approaches use measures of symptom duration (11/21), symptom severity (11/21) and/or the level of intervention required to manage the harm (14/21). However, few of these approaches account for the deleterious effects of hospitalization or the psychological stress that may be experienced by patients and/or their relatives. The new classification system we developed builds on WHO’s International Classification for Patient Safety and takes account not only of hospitalization and psychological stress but also of so-called near misses and uncertain outcomes. The constructs we have outlined have the potential to be applied internationally, across primary-care settings, to improve both the detection and prevention of incidents that cause the most severe harm to patients.

## Résumé

Les soins primaires ont du retard sur les soins secondaires en ce qui concerne l'établissement de rapports sur les incidents qui menacent la sécurité des patients et les enseignements qui en découlent. Dans le cas des soins primaires, il n'existe pas de méthode universellement acceptée pour classifier la gravité des dommages résultant d'incidents liés à la sécurité des patients. L'absence d'une telle méthode limite les enseignements qui pourraient favoriser la prévention des traumatismes chez les patients. Dans le cadre d'une analyse documentaire sur la sécurité des patients en matière de soins primaires, nous avons repéré l'existence de 21 méthodes de classification de la gravité des dommages. En prenant comme référence la Classification internationale pour la sécurité des patients de l'Organisation mondiale de la Santé (OMS), nous avons entrepris une analyse du cadre de ces méthodes. Nous avons ensuite conçu un nouveau système de classification de la gravité des dommages. Pour évaluer et classifier les dommages, la plupart des méthodes existantes utilisent des mesures portant sur la durée des symptômes (11/21), la gravité des symptômes (11/21) et/ou le niveau d'intervention requis pour prendre en charge les dommages (14/21). Néanmoins, rares sont celles qui tiennent compte des effets délétères de l'hospitalisation ou du stress psychologique que peuvent ressentir les patients et/ou leurs proches. Le nouveau système de classification que nous avons élaboré repose sur la Classification internationale pour la sécurité des patients de l'OMS et tient compte non seulement de l'hospitalisation et du stress psychologique, mais aussi de ce qu'il est convenu d'appeler les accidents évités de justesse et des résultats incertains. Les concepts que nous avons définis peuvent être appliqués dans les établissements de soins primaires du monde entier pour améliorer la détection et la prévention des incidents qui provoquent les plus graves dommages pour les patients.

## Resumen

La atención primaria queda por debajo de la atención secundaria en la notificación y el aprendizaje de incidentes que ponen en riesgo la seguridad del paciente. En la atención primaria, no existe un enfoque universalmente aceptado para clasificar la gravedad del daño que surge de tales incidentes que afectan a la seguridad del paciente. Esta falta de un enfoque consensuado limita el aprendizaje que podría conducir a la prevención de lesiones a los pacientes. En una revisión de la investigación sobre la seguridad del paciente en la atención primaria, se identificaron 21 enfoques existentes para la clasificación de la gravedad del daño. Con la Clasificación Internacional para la Seguridad del Paciente de la Organización Mundial de la Salud (OMS) como referencia, se llevó a cabo un análisis del marco de estos enfoques. A continuación, se desarrolló un nuevo sistema para la clasificación de la gravedad del daño. Para evaluar y clasificar el daño, la mayoría de los enfoques existentes usan medidas de la duración de los síntomas (11/21), la gravedad de los síntomas (11/21) y/o el nivel de intervención necesario para gestionar el daño (14/21). Sin embargo, pocos de estos enfoques explican los efectos nocivos de la hospitalización o el estrés psicológico que pueden experimentar los pacientes y/o sus familiares. El nuevo sistema de clasificación desarrollado se basa en la Clasificación Internacional para la Seguridad del Paciente de la OMS y tiene en cuenta no solo la hospitalización y el estrés psicológico, sino también los denominados casi accidentes y los resultados inciertos. Los constructos descritos tienen el potencial de aplicarse internacionalmente, en entornos de atención primaria, para mejorar tanto la detección como la prevención de incidentes que causan los daños más graves a los pacientes.

## ملخص

تتراجع العناية الأساسية عن العناية الثانوية في تقديم البلاغات بشأن الوقائع التي تعرض سلامة المريض للخطر واكتساب المعرفة منها. ولا يوجد أسلوب منهجي متفق عليه بشكل عام في مجال الرعاية الأساسية لتصنيف مدى خطورة الضرر الناشئ عن تلك الوقائع التي تتهدد سلامة المريض. ويحد هذا النقص في وجود أسلوب منهجي من إمكانات اكتساب المعرفة التي قد تقود إلى الوقاية من تعرض المرضى للإصابة. وفي مراجعة للأبحاث المتعلقة بسلامة المرضى في مجال الرعاية الأساسية، فقد حددنا 21 أسلوبًا منهجيًا قائمًا لتصنيف مدى خطورة الضرر. وباستخدام التصنيف الدولي لمنظمة الصحة العالمية لسلامة المرضى كمرجع، فقد اعتمدنا تحليلاً إطاريًا لتلك الأساليب المنهجية. ثم قمنا بتطوير نظام جديد لتصنيف مدى خطورة الضرر. ولكي يتم تقييم الضرر وتصنيفه، تستخدم أغلب الأساليب المنهجية الحالية مقاييس لمدة ظهور الأعراض (11/21)، وشدة الأعراض (11/21) و/أو مستوى التدخل المطلوب للتعامل مع الضرر (14/21). إلا أن القليل من تلك الأساليب المنهجية يتناول الآثار الضارة لاحتجاز المرضى في المستشفيات أو الضغط النفسي الذي قد يتعرض إليه المرضى و/أو ذووهم. ويعتمد نظام التصنيف الجديد الذي قمنا بتطويره على التصنيف الدولي لسلامة المرضى المعتمد لدى منظمة الصحة العالمية، ولا يقتصر تركيزه على احتجاز المرضى في المستشفيات فحسب والضغط النفسي الناشئ عنه، وإنما يتناول أيضًا ما يُطلق عليه حالات الضرر الوشيكة والمحصلات غير اليقينية. وهناك إمكانية لتطبيق التراكيب التي سلطنا الضوء عليها على المستوى الدولي وفي مختلف بيئات الرعاية الأساسية، وذلك لتحسين القدرة على رصد ومنع الوقائع الكفيلة بإيقاع أشد الضرر بالمرضى.

## 摘要

在将患者安全置于危险的事故报道和经验教训中，初级医疗落后于次级医疗。在初级医疗中，尚无广泛认可的方法来分类这些由患者安全事故所引发的伤害的严重程度。缺乏认可一致的方法限制了从中吸取教训，进而预防对患者的伤害。在一项针对初级医疗患者安全的研究回顾中，我们确认了 21 种现有的对伤害严重程度的分类方法。参照世界卫生组织国际患者安全分类，我们对这些方法进行了框架分析。之后，我们开发了一种伤害严重程度分类的新体系。为了对伤害进行评估和分类，大多数现有方法采用症状持续期 (11/21)、症状严重程度 (11/21) 和/或管理伤害所需的干预程度 (14/21) 来进行评估。然而，这些方法很少对住院治疗的有害影响和患者和/或家属可能经历的心理压力负责。我们开发的新分类体系建立在世界卫生组织国际患者安全分类的基础上，不仅考虑住院和心理压力，也考虑未遂事故和不确定结果。我们提出的构想有跨越初级医疗背景、广泛应用于国际领域的潜力，可以提升对患者造成的最严重伤害事故的检测和预防水平。

## Резюме

Первичная медико-санитарная помощь отстает от вторичной помощи в представлении и изучении информации об инцидентах, которые ставят под угрозу безопасность пациентов. В сфере первичной медико-санитарной помощи не существует общепризнанного подхода к классификации тяжести вреда, возникающего в результате инцидентов, связанных с безопасностью пациентов. Отсутствие согласованного подхода ограничивает возможность изучения информации об инцидентах, что могло бы способствовать предотвращению травматизма пациентов. В обзоре исследований по проблеме безопасности пациентов в сфере первичной медико-санитарной помощи авторы выявили 21 существующий подход к классификации тяжести вреда здоровью. Используя Международную классификацию Всемирной организации здравоохранения (ВОЗ) в области безопасности пациентов в качестве эталонной, авторы провели рамочный анализ этих подходов. Затем авторы разработали новую систему классификации тяжести вреда здоровью. Для оценки и классификации вреда здоровью большинство существующих подходов используют показатели продолжительности симптомов (11/21), тяжести симптомов (11/21) и (или) уровня вмешательства, требуемого при оказании медицинской помощи (14/21). Однако лишь некоторые из этих подходов учитывают негативные последствия госпитализации или психологического стресса, которые могут испытывать пациенты и (или) их родственники. Разработанная авторами новая система классификации основана на Международной классификации ВОЗ в области безопасности пациентов и учитывает не только госпитализацию и психологический стресс, но и так называемые потенциально опасные инциденты без последствий и неопределенные последствия. Описанные авторами конструкции потенциально могут применяться на международном уровне в рамках системы первичной медико-санитарной помощи для улучшения как обнаружения, так и предотвращения инцидентов, которые причиняют наиболее тяжелый вред здоровью пациентов.

## Introduction

Health organizations have a responsibility to learn from health-care-associated harm. In 2002, the World Health Assembly called for action to reduce the scale of preventable deaths and harm arising from unsafe care.^1^ Almost immediately, several health systems responded to this call. Most of these health systems had, at the core of their mission, a commitment to learn from medical errors and adverse events. Most subsequently set up systems to report and learn from so-called patient-safety incidents. One assumed that such systems would facilitate both the identification of systemic weaknesses that contribute to errors in health care and the learning necessary to prevent such errors recurring. However, in contrast to some other high-risk industries, where learning from accidents, mistakes and system failures appears to have led to major improvements in safety,^2^ little evidence exists that such systems have led to general reductions in the incidence or severity of patient-safety incidents.

If they have nationwide coverage systems for recording patient-safety incidents may receive very large numbers of reports each month. Within the United Kingdom of Great Britain and Northern Ireland, for example, there are about 100 000 reports of patient-safety incidents from England and Wales every month.^3^^,^^4^ Although the data collected on each incident have some value, it is not feasible to investigate so many incidents on an individual basis. A standardized, valid method of identifying the most important incidents is needed. There is international consensus that incidents leading to death or other severe consequences should be at the top of the list for analysis.^5^ However, the identification and valid prioritization of the more severe incidents is dependent on an accurate and consistent system for the classification of patient-safety incidents according to the severity of the harm that has occurred.

In the field of patient safety, much educational material comes from the narrative accounts of clinical staff reporting patient-safety incidents, and such accounts are a key component of many reporting systems.^6^ In 2009, the World Health Organization (WHO) developed the International Classification for Patient Safety.^7^ This classification, hereafter called WHO’s International Classification, was based on several earlier conceptual approaches to patient safety^8^^,^^9^ and potentially enables the international and inter-specialty comparison of incidents. The classification system defines five degrees of harm severity, from no harm to death (Box 1). However, it remains a work-in-progress and although it was developed for universal use, it is mostly based on the results of hospital-based research, even though more patients are seen in primary-care facilities than in hospitals.^10^ As primary care differs from hospital care in several important ways, e.g. in patient characteristics, organizational structure, relationships between health-care professionals and patients and types and outcomes of patient-safety incidents, the risks associated with hospital-based care should not be assumed to be the same as those associated with primary care.^10^^–^^12^

Box 1 **World Health Organization’s International Classification for Patient Safety: descriptions of harm severity^5^**

**None**

Outcome was not symptomatic or no symptoms were detected and no treatment was required.

**Mild**

Patient outcome was symptomatic, symptoms were mild, loss of function or harm was either minimal or intermediate but short-term and no intervention or only a minimal intervention, e.g. extra observation, investigation, review or minor treatment, was required.

**Moderate**

Patient outcome was symptomatic, required more than a minimal intervention, e.g. additional operative procedure or additional therapeutic treatment, and/or an increased length of stay and/or caused permanent or long-term harm or loss of function.

**Severe**

Patient outcome was symptomatic, required a life-saving or other major medical/surgical intervention, shortened life expectancy and/or caused major permanent or long-term harm or loss of function.

**Death**

On balance of probabilities, death was caused or brought forward in the short-term by the incident.

In 2016, the results of the then-largest analysis of patient-safety incidents during primary care were reported.^13^ These results indicated that the harm-severity element of WHO’s International Classification lacked sufficient granularity for the classification to be used in primary-care settings, especially when categorizing hospital admission, because of iatrogenic harm.^13^ By 2016, there was no universally-agreed system for the classification of harm severity in patient-safety incidents during primary care. The use of several different frameworks for assessing the severity of harm arising from patient-safety incidents in primary care had made the valid comparison of the relevant data from different countries very difficult.^10^

We therefore decided to investigate how the other classification frameworks used in this field aligned with WHO’s International Classification^7^ and then, by building on WHO’s International Classification, we developed a new, more comprehensive system for the classification of harm severity in patient-safety incidents occurring in primary care.

## Development of new system

### Systematic review

We searched the research literature for studies published before May 2016 that had used a framework, system or taxonomy for the classification of harm severity in patient-safety incidents occurring in primary care. Two reviewers searched 18 databases to find research studies and systematic reviews that had covered patient-safety incidents in primary care. Disagreements over inclusion were resolved via arbitration by a third reviewer. We based the searches on, and used the same search terms and methods as, a key systematic review,^10^ but brought that review up-to-date by including all relevant articles and grey literature published since 1980. We found 38 relevant studies and these covered 21 distinct systems for the classification of harm severity.^7^^,^^14^^–^^33^

### Framework analysis

We carried out a framework analysis of the content of each of the 21 classification systems, to identify the key themes and, particularly, each system’s strengths and weaknesses relative to WHO’s International Classification.^7^ We especially focused on the range of characteristics used to define harm. Encompassing both a-priori and emerging concepts, framework analysis facilitates the development of a themed matrix by organizing and managing data through a process of summation.^34^

### System development

We used the results of the framework analyses in developing our new classification system, through an iterative process. Together, we have experience in coding and analysing over 60 000 reports of patient-safety incidents in primary care for several mixed-methods research studies.^13^^,^^35^^–^^39^

We discussed the common and novel features of each classification system and considered the practicalities of identifying such characteristics when coding incidents that had occurred during primary care. Finally, we tested the classification system iteratively, by applying it to randomly generated samples of 100 incident reports, revising the system by clarifying the definitions and then applying the revised system to further samples of reports. We discussed the revised definitions and the reasons for changes until we had a consensus.

### Terminology

The results of the systematic review revealed that there were several common features within the 21 existing approaches to the classification of harm in patient-safety incidents during primary care. Most of these approaches involved harm categories that ranged from death at one extreme (16/21) to no harm, or an equivalent synonym, at the other (12/21). Eleven of the approaches used another three descriptive categories of harm, that is, either mild, moderate and severe or synonyms of these adjectives, like WHO’s International Classification.^7^ We decided to use the same categories in our new classification as they were relatively intuitive and globally understood (Table 1). The other 10 approaches used a grading systems based on letters or numbers, such as that used by the United States of America’s National Coordinating Council for Medication Error Reporting and Prevention.^15^

**Table 1:** Primary Care Harm Severity Classification System, 2018

Severity	Definition	Examples
No harm	Any incident that ran to completion but no harm occurred to the patient	Patient received azathioprine but missed routine haematological monitoring for several months. No harm incurred
No harm outcome due to mitigating action	Any incident that had the potential to cause harm to a patient but resulted in no harm	A receptionist issued an incorrect prescription that indicated a patient should take one tablet twice daily instead of once daily. The chemist providing the tablets, who had dispensed to the patient previously, noted the error and corrected the regimen
Mild harm	Incident in which: (i) patient was harmed, with mild and short-term impact, on physical, mental or social functioning, that was expected to resolve in a few hours; (ii) patient was harmed but required no or minimal intervention/treatment, e.g. anti-emetic, oral antibiotic or repeat of a minor procedure such as vaccination or insertion of contraceptive implant; and/or (iii) patient or their loved ones experienced transient emotional distress but no long-term consequences and incident report contains words, e.g. angry, anxious, confused, distressed, frightened, frustrated, humiliated or upset, that might describe a feeling that occurs at the time of the incident but soon passes	An on-call primary-care physician prescribed oral analgesic for a patient who could not swallow. A second physician also made a prescription error, leaving patient in pain for three hours. Relatives of a patient dying at home were unable to get drugs for a syringe driver at a weekend because their local pharmacy was out of stock. Their local hospital would not supply the drugs but they were eventually obtained from a community health-care provider. The patient was left without drugs for 3.5 hours and the relatives were very distressed
Moderate harm	Incident in which: (i) patient was harmed, causing a medium-term impact on physical, mental or social functioning that was expected to resolve in days; (ii) patient required medical intervention in the form of treatment, e.g. antibiotics or intravenous fluids; (iii) patient required short-term hospitalization for assessment and/or minor treatment in either ED or a hospital ward; and/or (iv) patient or their loved ones experienced psychological difficulty of a more longstanding nature but not requiring formal treatment, e.g. as indicated by evidence in the report of more longstanding anxiety, insomnia, or low mood	A health-care provider made a routine visit to a diabetic patient to administer insulin. The patient’s blood sugar was found to be within safe limits to administer insulin and insulin was therefore given. Later on the same day, the patient was found to be hypoglycaemic. It was discovered that the patient, who had learning difficulties, had failed to tell the provider that he had received insulin 30 minutes before the provider’s visit. He was admitted to a local hospital for monitoring of blood sugars overnight. A patient was prescribed amoxicillin despite being known to have penicillin allergy. Although the error was corrected and the patient given clarithromycin, the patient claimed to have lost trust in doctors and to be extremely anxious about taking the clarithromycin
Severe harm	Incident in which: (i) patient was harmed, causing a major long-term or permanent impact on physical, mental or social function or shortening of life-expectancy; (ii) patient was harmed and required major medical or surgical intervention that, most often, was delivered in a hospital setting, e.g. cardioversion, any major surgery; (iii) patient was harmed and required prolonged hospitalization or admission to CCU, HDU and/or ICU; and/or (iv) patient or their loved ones experienced enduring psychological difficulty that required specialist treatment, e.g. as indicated in the report by evidence of chronic anxiety or depression or psychosis	An epileptic child who had been prescribed phenobarbital was admitted with symptoms of drowsiness and had decreased tone for three days. He was ventilated and immediately transferred to the ITU because he had a low GCS score. His blood concentration of phenobarbital was found to be abnormally high. When the patient’s own supply of phenobarbital was checked, the original manufacturer’s label gave the strength as 25 mg/mL but the erroneous community pharmacy’s label indicated 25 mg/5 mL. The child had been receiving five times the prescribed dose
Death	Incident in which, on the balance of probabilities, death of the patient was caused or brought forward in the short term by the incident	A patient contacted an out-of-hours service by telephone, reporting feeling unwell, vomiting and a rash on his stomach. A physician, who returned the patient’s call, diagnosed a viral illness and asked the patient to make arrangements for a relative to collect a prescription for an anti-emetic. Within 90 minutes, however, the patient had deteriorated and been brought to the ED of his hospital. The patient was diagnosed with meningococcal septicaemia and died
Insufficient detail	Incident for which the report carries insufficient information to evaluate the severity of harm. The report may describe an error or outcome that was not the result of primary health care, e.g. a fall in the waiting room. Alternatively, it may fail to describe any outcome or it may describe a patient-safety incident but give insufficient information to classify the severity of harm of the outcome, e.g. it may record a delay in getting an appointment but not describe the consequences of the delay for the patient	A patient provided samples for histology and cytology, but the provider collecting the samples in specimen pots forgot to label the pots

ED: emergency department; CCU: coronary care unit; GCS: Glasgow coma scale; HDU: high dependency unit; ICU: intensive care unit; ITU: intensive therapy unit.

### Ways of defining harm

The results of the systematic review revealed that most of the 21 existing approaches had been based on at least one of three broad parameters: (i) the severity of the symptoms or loss of function (11/21);^7^^,^^14^^,^^17^^,^^21^^,^^23^^–^^25^^,^^28^^,^^30^^,^^31^^,^^33^ (ii) the duration of the symptoms (11/21);^7^^,^^14^^–^^16^^,^^18^^,^^20^^,^^23^^–^^25^^,^^31^^,^^33^ and/or (iii) the interventions required, e.g. investigation, treatment and/or hospitalization, as a result of the incident (14/21).^7^^,^^14^^–^^19^^,^^21^^–^^24^^,^^30^^,^^32^^,^^33^ Three of the 21 approaches were relatively simple and did not use any of these parameters.^26^^,^^27^^,^^29^

In our new classification, we included all three broad parameters for defining physical harm because there is wide diversity in the types of incidents and descriptions of outcomes that occur in primary care. To define the severity of a patient’s symptoms and/or loss of function, we used the term “impact on physical, mental or social functioning”, which is applicable to a wide range of cultural settings and conforms with the terms used by WHO for the assessment of quality of life.^40^ As an outcome of a patient-safety incident, we found pain difficult to categorize as it is subjective and affected by factors such as: the patient’s environment, their mood and their understanding of cause and prognosis.^41^ We ask users of our new classification system not to make assumptions about the severity of the symptoms, e.g. by recording all pain as “mild harm” but, instead, to concentrate on objective features e.g. the duration of the symptoms and their impact on the patient’s mental, physical and social functioning.

### Hospitalization

Although our systematic review revealed 12 relevant approaches to harm classification that identified hospital admission as a key marker of severity, the 12 varied in the grade of severity that they allocated to hospitalization. In WHO’s International Classification,^7^ “increased length of stay” equates to “moderate harm.” Of the primary-care-specific approaches that we identified, four and two considered hospitalization to be indicative of severe^17^^,^^21^^,^^23^^,^^30^ and moderate harm,^19^^,^^32^ respectively. For patient-safety incidents in primary care, we think that the full impact of hospitalization on the patient and the health-care provider, both financially and in terms of the mental, physical and social costs, should be appreciated.^42^ Not all hospitalizations represent harm of the same severity. For example, compared with an admission to a high-dependency unit for several weeks, an admission to an emergency department for a few hours of observation or minor treatment is clearly indicative of a less severe form of avoidable harm. We decided that, in our new classification system, we would distinguish these two admissions as indicative of severe and moderate harm, respectively.

### Psychological harm

WHO’s International Classification uses only physical health outcomes to classify harm severity.^7^ However, for the patient involved, the psychological stress associated with a patient-safety incident can often have a greater impact than any physical harm.^43^ Although our systematic review revealed 21 existing approaches to the classification of harm in patient-safety incidents during primary care, only six of these approaches took psychological outcomes, described as emotional, mental or psychological harm, into account.^18^^,^^20^^,^^24^^,^^25^^,^^30^^,^^31^ Just two approaches enabled the classification of moderate or severe psychological harm.^20^^,^^31^ One approach described emotional injury as a low-severity category^25^ while three ranked psychological harms between their no-harm and mild-harm categories.^18^^,^^24^^,^^30^

In general, health-care professionals intuitively recognize emotional harm to patients and seek to avoid such harm. However, those who report patient-safety incidents may neglect psychological outcomes in their reports^44^ and failures to report such outcomes may limit our understanding of the true nature of health-care-associated harm. Our new classification system encompasses mild, moderate and severe psychological harm as well as physical harm outcomes. We considered “emotional harm” to be the most appropriate terminology for mild, and generally transient, harm but used “psychological harm” to describe moderate or severe and, usually, more enduring harm.^45^

Many incident reports describe how the affected patients and/or their families were distressed by an incident. From the evidence that was routinely reported, we decided that a group of people who had struggled to obtain medications for a dying relative (Table 1) should be considered to have suffered only mild emotional harm. Although we thought that this event must have been extremely upsetting and is unlikely to be forgotten over the long term by the family involved, a key principle in our approach is that nothing that is not explicitly stated in an incident report should be inferred.^13^ In the future, we anticipate that our new classification system will be used by frontline health-care professionals and risk managers who, when struggling to evaluate the level of psychological harm, will often be able to obtain clarification after more detailed investigation of an incident.

### Near misses

WHO’s International Classification does not allow incidents where there was no risk of harm to be distinguished from so-called close-call or near-miss incidents, that is, incidents where there was no harm only because harm was prevented by a timely safety intervention. However, our systematic review revealed several approaches that permit such distinction.^14^^,^^15^^,^^17^^,^^30^^,^^33^ For example, one of these approaches differentiated between “no harm, impact prevented” and “no harm, impact not prevented”.^14^ The taxonomies developed by the United States National Coordinating Council for Medication Error Reporting and Prevention^15^ and Linnaeus-PC Collaboration^18^ each have four separate no-harm categories that indicate whether the potentially harmful outcome reached the patient and whether an intervention took place to prevent a harmful outcome occurring. As reports of near-miss incidents should help health-care providers to learn how to prevent or, at least, reduce harm,^46^^,^^47^ we made a separate category to capture such incidents a key component of our new classification system.

### Uncertainty

At the time that a patient-safety incident is reported, the eventual outcome for the patient may be unknown. Reporting systems and classifications must allow for this uncertainty. We found that, of the 21 approaches investigated in our systematic review, eight allowed for a degree of uncertainty about the outcome. For example, each of the definitions used in one approach is prefaced with *“*error occurred that might have contributed to or resulted in harm”.^15^ The taxonomy produced by the Linnaeus-PC Collaboration contains a specific category to cover incidents where “an error occurred, but it was not possible to determine harm”.^18^ Our new classification includes an unknown-harm category, partly to cover events where the reported harm outcomes cannot be unequivocally attributed to the reported incidents.

## Finalizing the new system

We named our new system the Primary Care Harm Severity Classification System. Table 1 provides examples, from reports collected by the United Kingdom’s National Reporting and Learning System, of patient-safety incidents that would be assigned to each of the new system’s categories of harm severity. To ensure confidentiality, we anonymized all of the reports that we used and removed date and/or location data. We used the insights gained from the process of applying the new classification system to real examples of patient-safety incidents to inform the concepts and definitions used in the new system (Box 2), which offer guidance to future users of the system.

Box 2 **Concepts and definitions used in the new Primary Care Harm Severity Classification System, 2018**

**Delayed diagnosis**

In cases of delayed diagnosis or treatment, the delay itself does not inform the severity. Instead, the severity score should be based on the outcome of the delay, if known, e.g. two months of additional pain due to a delayed diagnosis should be coded as moderate harm due to the duration of pain.

**Harm**

In the system, harm is considered to be the impairment of structure or function of the body and/or any deleterious effect arising from, or associated with, plans or actions taken during the provision of primary health care. It includes disease, injury, suffering, disability and death and may be physical, psychological or social.

**Incident**

In the system, an incident is an event or circumstance that could have resulted, or did result, in unnecessary harm to a patient.

**Inconvenience to the patient**

The system makes no specific reference to inconvenience but, where appropriate, a patient’s frustration could be understood as emotional harm or in terms of the physical harm caused, e.g. increased duration of symptoms, and classified accordingly.

**Mitigating action**

A mitigating action could be by anyone, including health-care professionals, patients or their relatives, e.g. a patient may notice an incorrect prescription and return the incorrect medication to a pharmacy, without taking it. Even reports of incidents in which there has been no harm due to mitigating action provide useful lessons in preventing harm.

**Outcome**

In the system, an outcome represents the impact upon a patient that is wholly or partially attributable to an incident.

**Uncertainty**

If it is clear that an incident caused harm, but the full severity of that harm cannot be assessed, the incident should be coded according to the least severe harm that is evident. Users of the system should avoid coding according to how they imagine the patient or the patient’s relatives might feel. If they are unable to discover any more detail of the incident, they should stick to the known facts. If users know that the relatives were angry about the incident, but not how long the anger lasted, the anger should be coded as mild harm.

**Unnecessary interventions**

An intervention or hospitalization resulting from an incident should be coded as harm even if it was unnecessary, e.g. a patient sent to an emergency department because the out-of-hours service was busy, would still be considered to have suffered moderate harm.

## Policy, practice and research implications

Definitions of harm severity vary greatly between existing classification systems for patient-safety incidents in primary care. In general, the adverse effects of hospitalization and psychological harm have previously been neglected. Health-care organizations need a consistent and reliable way of knowing which aspects of their care result in the most harm to patients. To help health-care teams to learn from patient-safety incidents, WHO’s Minimal Information Model for Reporting Patient Safety Incidents encourages the use of a standardized essential data set, with harm severity as a key component.^48^ Advances in the methods of analysis of incident reports from primary-care facilities should facilitate the scoping of action to reduce risk and improve patient safety, including the planning for research that could lead to more effective interventions.^13^

Our new classification system for harm severity is a starting point for a learning process that should lead from the more effective analysis of reports on patient-safety incidents, to the prevention of such incidents and the associated harm, in the future. Health systems already operating or developing systems for the reporting of patient-safety incidents that are compatible with WHO’s International Classification^7^ should find our classification system relatively easy to apply, since our system is builds on WHO’s International Classification. If applied universally, our new classification system will allow temporal and geographical comparisons of the severity of patient-safety incidents occurring in different primary-care systems.

## Next steps

The effective application of any system for classifying the severity of harm associated with patient-safety incidents depends on judgments made by the individuals coding the incidents. Such judgements will vary depending on each coder’s clinical role, level of clinical knowledge and past experiences. In this paper, we have mapped out the key constructs for inclusion in an appropriate framework for classifying the severity of harm associated with patient-safety incidents in primary care. Given the broad range of events described in incident reports, the wide scope of the definitions we use is intentional. Although a lengthier and more prescriptive classification system may achieve greater consistency between users, it risks being too complex to use in practice and too reductionist to support useful interpretation and learning. In the future, we plan to undertake a validation study in which a diverse, multidisciplinary panel of primary-care professionals, researchers and patient advocates will be asked to use the new classification system, initially to code examples of reports of patient-safety incidents recorded in the United Kingdom’s National Reporting and Learning System. From our experience of applying classification systems in multiple contexts, we recognize that the users of such systems must be able to select codes with intuitive definitions that the users understand and find relevant to their work. Stakeholders may wish to adapt the classification system to support maximal learning in their local settings. However, in the interests of national and international learning and maximizing opportunities to learn from rare events, the key constructs we outline must be consistently applied. Each organization applying the new classification system must ensure comprehensive training is provided for key stakeholders. If users can be kept informed of the value of their coding, they may provide increasingly meaningful incident reports in the future.

## Conclusions

Previous attempts to identify and learn from the most important sources of harm to patients in primary care have been restricted by the lack of a universal standard system for classifying the severity of such harm and the general neglect of psychological harm in this context. Health-care leaders must develop robust mechanisms for generating useful reports of patient-safety incidents and acting on those reports to improve patient safety. We have empirically developed a new classification system that has the potential to be applied internationally, across primary-care settings, to improve the detection and prevention of incidents that cause the most severe harm to patients.
